# Studies on the antimicrobial potential and structural characterization of fatty acids extracted from Sydney rock oyster *Saccostrea glomerata*

**DOI:** 10.1186/s12941-014-0057-x

**Published:** 2014-12-31

**Authors:** Subbiahanadar Chelladurai Karthikeyan, Subramanian Velmurugan, Mariathason Birdilla Selva Donio, Mariavincent Michaelbabu, Thavasimuthu Citarasu

**Affiliations:** Centre for Marine Science and Technology, Manonmaniam Sundaranar University, Rajakkamangalam, Kanyakumari, 629502 Tamilnadu India

**Keywords:** Antimicrobial factors, Antitumor, Fatty acids, *Saccostrea glomerata*

## Abstract

**Background:**

The marine environment having vast resources of natural products with potential bioactivities. Among the marine natural products, fatty acids obtained from marine mollusks have broad range of biological activities including antimicrobial and antitumor activities. The present study aims to characterize the fatty acid derivatives from the Sydney rock oyster *Saccostrea glomerata* and its pharmacological activities*.*

**Methods:**

*S. glomerata* fleshes were serially extracted with hexane, ethyl acetate and methanol and studied the antimicrobial activities against pathogenic bacteria, fungi and virus. Based on the better result, the ethyl acetate extract was selected and purified through silica column chromatography and screened the fractions for antimicrobial and antitumor activities. Also the best active fraction (FV) was functionally and structurally characterized.

**Results:**

The ethyl acetate extract of *S. glomerata* effectively controlled the bacterial pathogens and formed of more than 15 mm of zone of inhibition and also effectively suppressed the fungal growth and inhibit the shrimp white spot syndrome virus (WSSV). The secondary screening results revealed that, the fraction (FV) had potential antimicrobial and antitumor activities. The FV concentration (100 μg/ml) effectively suppressed the tumor mammary epithelial carcinoma cell of 14.45%. The GC–MS analysis revealed that, eleven compounds including N-hexadecanoic acid, L-(+)-ascorbic acid 2,6-dihexadecanoate and 6-Octadecenoic acid were characterized.

**Conclusions:**

The fatty acid derivatives isolated and characterized from *S. glomerata* extracts had the potent antimicrobial and antitumor activities. This basic research can help to develop the antimicrobial and anticancer drugs from the nutraceuticals in future.

## Introduction

Mollusks are widely distributed throughout the world and have many representatives such as slugs, whelks, clams, mussels, oysters, scallops, squids and octopods in the marine and estuarine ecosystem [1]. Filter-feeding bivalves like oysters are benthic organisms and are constantly exposed to relatively high concentrations of bacteria, viruses, parasites, and fungi many of which may be harmful to the organism. The survival of these organisms might depend on efficient antimicrobial mechanisms to protect themselves against microbial infections. In addition, their immobile life style might increase the risk of pathogenic infection. To combat these potential invaders, they need an effective defense system [2].

The marine environment is a huge source to discover bioactive natural products. A wide variety of bioactive substances are being isolated and characterized from the food that is derived from the marine environment, several with great promise for the treatment of human and fish disease. For the past two decades, pharmaceutical industry has been relatively successful in overcoming problems due to single resistant determinants; however the advent of multiple resistant mechanism has severely limited the use of many major classes of antimicrobial compounds [3]. In marine invertebrates so far approximately 7000 marine natural products have been reported, 33% from sponges, 18% from coelenterates and 24% from representatives of other invertebrate phyla such as ascidians, mollusks, echinoderms and bryozoans [4]. However, the advent of multiple resistant mechanism has limited the use of many major classes of antimicrobial compounds. The bio prospecting of novel natural compounds from marine invertebrates may be effective, non-toxic and highly promising to the antimicrobial research.

Several compounds extracted from marine invertebrates, including bivalves, possess broad spectrum antimicrobial activities, affecting the growth of bacteria, fungi and yeasts [5,6]. Antibacterial and antiviral activities have been previously described in the hemolymph of several molluscan species including several sea hares, sea slung, oysters and mussels’ species [7-10]. Oyster extract has many biological activities including antitumor and anticancer [11], antioxidants [12], antibacterial [13], antiviral [14] and reducing blood pressure [15]. The fatty acids obtained from marine mollusks have broad range of biological activities. The ability of fatty acids to interfere with bacterial growth and survival has been known for several decades [16]. Bioactive lipids from mussels including fatty acids, sphingolipids, phytosterols, diacylglycerols, diterpenes, sesquiterpenes and saponins were highly influenced to control the human diseases [17]. The present study intends to characterize the lipid compounds extracted from Sydney rock oyster *Saccostrea glomerata* and to understand the antimicrobial and anticancer activities *in vitro* level.

## Materials and methods

### Sampling and extraction

Live specimens of the Sydney rock Oyster *Saccostrea glomerata* was collected from Kovalam rocky shore (8° 4’ 38.39” N; 77° 31’ 56.32” E) of Kanyakumari district, Tamilnadu, India. They were immediately brought to the laboratory and their soft bodies were removed by breaking shells. The fleshes were cut into small pieces, washed with distilled water and homogenized. The homogenate was serially extracted with three times of hexane, ethyl acetate and methanol. The extract was filtered and concentrated in a rotary vacuum evaporator at 40°C and stored at 4°C.

### Determination of antimicrobial activity

#### Antibacterial screening

*In vitro* antibacterial activity was performed by the extracts taken from *S. glomerata* using different organic solvent and condensate to the total volume of 50 μg. The extracts were tested against aquatic and human diseases causing bacterial pathogens including *Pseudomonas aeruginosa, Vibrio harveyi, V. parahaemolyticus, Aeromonas hydrophila* and *Staphylococcus aureus* using agar diffusion following the method of Baur et al. [18].

#### Antifungal screening

For antifungal activity, *Aspergillus niger, A. flavus, Candida albicans* and *Fusarium* sp. spores (1× 10^8^ cfu^−1^) were inoculated onto sabouraud dextrose agar. Sterile cork borer was used to bore five holes on the agar plates and then the *S. glomerata* extracts were introduced aseptically and incubated at 30°C for 5 days. The zone of inhibition was recorded.

#### Antiviral screening

Antiviral activity was performed against the whispovirus, White Spot Syndrome Virus (WSSV) infected shrimps. The haemolymph samples of the infected shrimp *Fenneropenaeus indicus* were bled and centrifuged at 3000 X g for 20 min at 4°C. Then the supernatant fluid was re-centrifuged at 8000 X g for 30 min at 4°C and the final supernatant fluid was filtered, the filtrate was then stored at – 20°C for infectivity studies. 50 mg of *S. glomerata* extracts condensate were dissolved in 10 ml of NTE buffer (0.2 M NaCl, 0.02 M Tris–HCl and 0.02 M EDTA and adjusted pH 7.4) as stock for further bioassay studies. 5 μl of viral suspension were mixed with 10 μl of *S. glomerata* extracts and incubated at 29°C for 3 hours. After 3 hours, the mixture was injected intramuscularly into shrimp *F. indicus* weighing 8.0 ± 1 g. Control experiments were also maintained as for the mixture of 25 μl NTE buffer and 5 μl viral suspensions. Mortalities were recorded in every day and the experiment was carried out up to 10 days.

### Purification of antimicrobial active extracts

Based on the best result, ethyl acetate extract of *S. glomerata* was purified through preparative silica column chromatography (50–80 μm particle size; 30 cm column length; 0.5 ml elution flow rate and three bed volume elution). Different proportions of the mobile phases such as hexane/ethyl acetate and ethyl acetate/methanol were used for eluting the active compounds. The different elutes were collected, concentrated in a rotary evaporator and stored at 4°C. The fractions were spotted on silica gel plates GF254 (Merck), 20 × 20 cm, 1 mm thick and the chromatogram was developed using, hexane: ethyl acetate (8:2) as mobile phase. The plates were visualized under short UV wavelength.

### Secondary antimicrobial screening

The antibacterial activity was performed against the bacterial pathogens including *P. aeruginosa, V. harveyi* and *A. hydrophila* using the *S. glomerata* extract fractions FIII, FIV, FV, FIX and FX following the method mentioned in the section 2.2.1. The antifungal activity was performed against the fungal pathogens including *A. niger, C. albicans* and *Fusarium* sp. following the method mentioned in the section 2.2.2. Antiviral activity was also performed against WSSV using the purified fractions following the method mentioned in the section 2.2.3. The haemolymph samples were collected from the challenged shrimps, extracted the genomic DNA and performed the WSSV diagnostic double step PCR described by Takahashi et al. [19].

### Antitumor assay

The antitumor assay was performed in tumor mammary epithelial carcinoma cell lines with different concentrations (10–100 μg/ml) of purified *S. glomerata* extract fraction (FV) following the method of Freshney [20] and the activity was monitored after 48 h.

### Functional group characterization by FT-IR spectroscopy

The basic functional groups of the purified *S. glomerata* column elution (FV) were analyzed qualitatively by Fourier Transform Infra Red (FTIR) method described by Kemp [21].

### Structural characterization by GC-MS analysis

GC–MS analysis of purified *S. glomerata* extract fraction (FV) was analyzed using Agilent GC–MS 5975 Inert XL MSD (United States) gas chromatography equipped with J and W 122–5532G DB-5 ms 30 × 0.25 mm × 0.25 μm and mass detector (EM with replaceable horn) that operated in EMV mode. Helium was used as carrier gas with the flow rate of 1.0 ml min^−1^. The injection port temperature was operated at 250°C. The column oven temperature was held at 80°C for 2 min then programmed at 10°C min^−1^ to 250°C, which was held for 0 min, and then at 5°C min^−1^ to 280°C which was held for 9 min. Electron impact spectra in positive ionization mode were acquired between *m/z* 40 and 450.

### Data analysis

One-way and two-way ANOVAs were carried out using the SPSS statistics data package and Ky plot, respectively. Positive correlation analysis also performed to compare the data by Microsoft Excel programme. Means were compared at 0.05and 0.001% level.

## Results

### Primary antibacterial and antifungal screening

Among the three organic solvent extracts of *S. glomerata,* the ethyl acetate extract (50 μg) was effectively suppressing the bacterial pathogens such as *P. aeruginosa, V. harveyi, V. parahaemolyticus, A.hydrophila* and *S.aureus* of more than 15 mm of zone of inhibition. The same extract also effectively controlled the fungal pathogens including *A. niger, A. flavus, C.albicans* and *Fusarium* sp. by *in vitro* level of antifungal screening (data not given).

### Primary antiviral screening

**Figure 1:**
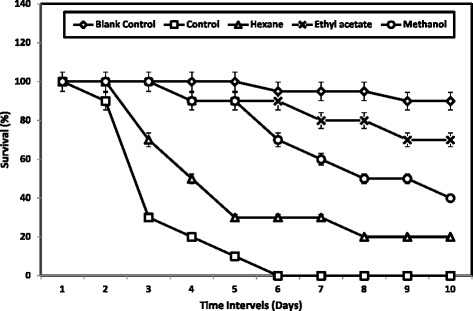
**Cumulative mortality of** ***F. indicus*** **injected with WSSV-incubated** ***S. glomerata*** **extracts.** The values are significantly differed from each other (*F =* 32.16; P <= 0.001) - Two way ANOVA.

The antiviral effect of the *S. glomerata* extract against White Spot Syndrome Virus (WSSV) revealed that, the ethyl acetate extract effectively suppressed the growth/pathological effect of WSSV. The WSSV injected shrimp in the control group succumbed to death within 6 days of post inoculation. The hexane extract treated group had 22% survival; methanolic extract treated group had 42% survival and ethyl acetate treated group had 70% survival respectively. Two way ANOVA revealed that, the survival values are significantly (*F* = 32.16; P ≤ 0.001) differed each other’s (Figure 1).

### Secondary antibacterial and antifungal screening

Among the different fractions eluted by column purification, the fractions including FIII, FIV, FV, FIX and FX were able to control the bacterial and fungal pathogens in *in vitro* screening methods. The fractions FV had controlled the pathogens and formed more than 14 mm of zone of inhibition. There were five spots (R_f_ of 0.29, 0.42, 0.57, 0.71) detected by TLC analysis in the fraction FV. This zone of inhibition observed was 16, 14.9 and 14.5 mm against *P. aeruginosa, V. harveyi* and *A. hydrophila* respectively by the FV fraction treated agar plates. The FIII and FX had little antibacterial activates. One way ANOVA revealed that the antibacterial results among the different fractions treated bacterial pathogens significantly (P < = 0.001) differed each others. The FV fraction also effectively suppressed the growth of fungal pathogens including *A. niger, C. albicans* and Fusarium sp. at *in vitro* level screening (Table 1).

**Table 1:** **Lists of active compounds with Rf values and their antimicrobial activities of the ethyl acetate extract of** ***S. glomerata*** **after column purification**

**Active fractions**	**Mobile phase**	**Spots with R**_**f**_ **values**	**Antimicrobial activities**					
			**Antibacterial (mm of zone of inhibition)**			**Antifungal activity**		
			***P. aeruginosa***	***V. harveyi***	***A. hydrophila***	***A. niger***	***C. albicans***	**Fusarium sp.**
F III	H 10: EA 40	0.39, 0.62, 0.77, 0.82	4.3 ± 0.2^a^	3.9 ± 0.15^a^	5.0 ± 0.15^a^	*-*	*-*	-
FIV	H 20: EA 30	0.21, 0.28, 0.40	11.3 ± 0.25^b^	10.6 ± 0.2^b^	9.5 ± 0.15^b^	+	+	++
FV	H 25 EA 25	0.29, 0.42, 0.57, 0.71	16.0 ± 0.20^c^	14.9 ± 0.18^c^	14. 5 ± 0.13^c^	++	++	++
F IX	EA 10: M 40	0.28, 0.36, 0.48, 0.56,0.73	13.8 ± 1.13^d^	9.4 ± 0.22^b^	7. 6 ± 0.25^d^	+	++	+
FX	EA 15 M 35	0.59, 0.79	4. 1 ± 0.12^a^	3.8 ± 0.17^a^	5. 1 ± 0.15^a^	-	-	-

H: Hexane; E: Ethyl acetate; M: Methanol.

++: Higher activity; +: Less activity; −: No activity.

Means with the same superscript do not significantly (P < = 0.001) – One Way ANOVA.

### Secondary antiviral screening

**Figure 2:**
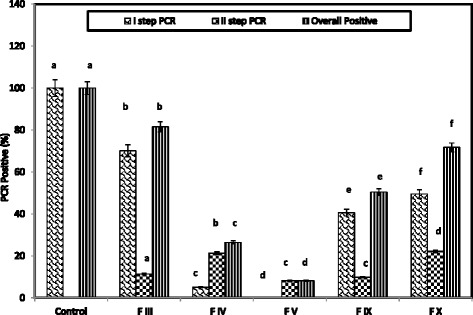
**PCR amplification using WSSV VP28 primer from the genomic DNA template of** ***F. indicus*** **injected with** ***S. glomerata*** **column fractions incubated WSSV.** Means with the same superscript do not significantly (P <= 0.001) – One Way ANOVA.

Double step PCR diagnosis for WSSV in controland all the fractions treated groups were given in Figure 2. All shrimps from the control group wereWSSV positive in the first step PCR detection. The infection was significantly decreased to 70.13, 49.53, 40.65, 5.15 and 0% in shrimps treated with FIII, FX, FIX, FIV and FV respectively (P < = 0.0001- one way ANOVA test). In the second step detection also, among the different fractions treated groups, the same trend was observed. The overall result indicated that the infection was decreased significantly (P < = 0.0001) to 81.53, 71.73, 50.45, 26.48 and 8.15% in FIII, FX, FIX, FIV and FV respectively. The FV fraction helps to reduce the infection of 91.85% from the control group.

### Antitumor activity

**Figure 3:**
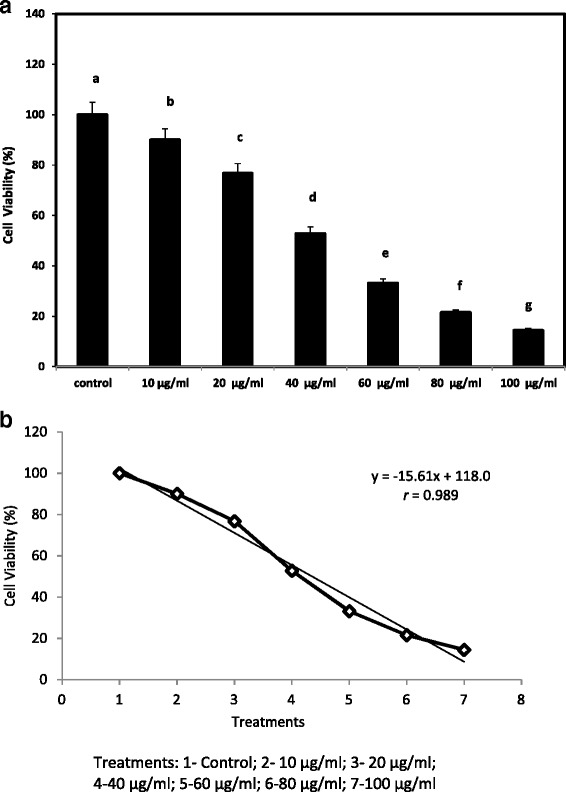
**a Anti tumor activity performed in tumor mammary epithelial carcinoma cell lines with different concentration of the FV fraction of** ***S. glomerata.*** Means with the same superscript do not significantly (P <= 0.001) – One Way ANOVA. **b** Positive correlation between control and *S. glomerata* FV fraction treated tumor mammary epithelial carcinoma cell lines*.* Values are the mean of three determinations ± SEM (P<0.05).

The cell viability of 100% was observed when no fatty acid derivatives treated tumor cells whereas the cell viability was gradually decreased with increasing the concentration on the treated tumor cells. The viability observed were 90.02, 76.8, 52.83, 33.16, 21.54 and 14.45%, in 10, 20, 40, 60, 80 and 100 μg/ml of purified fatty acid derivatives of FV treated tumor cells. One way ANOVA revealed that, the antitumor activity results among the different concentrations treated cells were significantly (P < = 0.001) differed each other’s (Figure 3a). The positive correlation analysis also revealed a significant relationship (P < 0.05) was found among the treatment from control to different concentration experimental groups (Figure 3b).

### Functional group analysis

**Figure 4:**
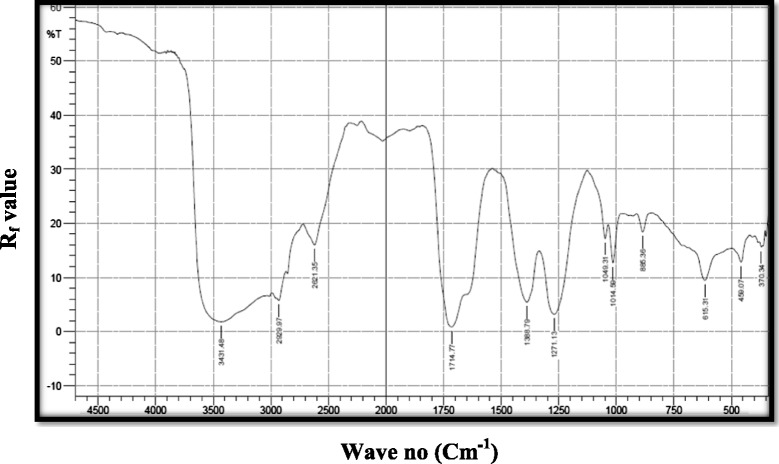
**FTIR analysis of the FV fraction of** ***S. glomerata*** **for functional group analysis.**

The peak around 3431.48 cm^−1^ may be due to hydrogen bonded –OH.The peak at 1049.31 cm^−1^ may be due to N-O group which is further supported by the peak at 1271.13 cm^−1^was due to N-O group and also peak at 1049.31 cm^−1^was also due to R_2_SO group. The peak at 1714.77 cm^−1^ may be due to a pure carboxyl group.The other peak around of 2929.97 cm^−1^ may be due to OH stretching. The other peak, one at 885.36 cm^−1^may be due to alkenes (Figure 4).

### Structural characterization

**Figure 5:**
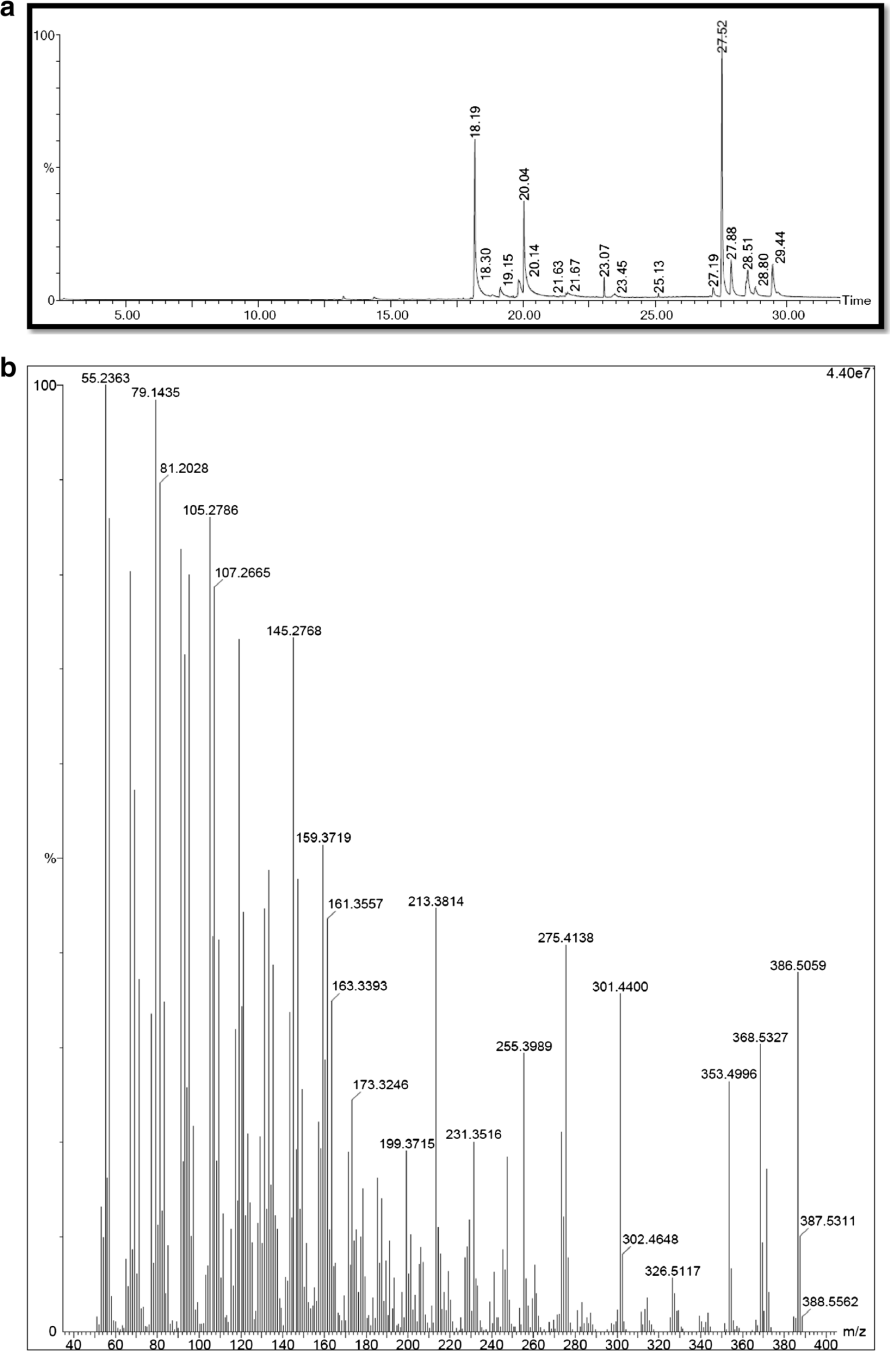
**a GC analysis of the FV fraction of** ***S. glomerata*** **for structural characterization.** **b** Mass spec analysis of the FV fraction of *S. glomerata* for structural characterization.

The GC–MS analysis (Figure 5a and b) characterized eleven lipid derivatives that had the antimicrobial and antitumor properties. The peak with at the retention time of 18.19 was confirmed as N-hexadecanoic acid with the molecular weight of 256 and formula of C_16_H_32_O_2_. The peak with the retention time of 19.15 was confirmed as L-(+)-ascorbic acid 2,6-dihexadecanoate with the molecular weight of 652 and formula of C_38_H_68_O_8._ The peak at the retention time of 19.83 was confirmed as 6-Octadecenoic acid which had the molecular weight of 282 and formula of C_18_H_34_O_2._ The peak with the retention time of 27.52 was confirmed as Cholesterol with the molecular weight of 386 and formula of C_27_H_46_O_._ The peak with the retention time of 28.79 was confirmed as stigmasterol which had the molecular weight of 412 and formula of C_29_H_48_O (Table 2).

**Table 2:** **Compounds identified from the FV fraction of** ***S. glomerata*** **using GC-MS analysis**

**RT**	**Compounds**	**Molecular formula**	**Molecular weight**	**Quality**	**Molecular structure**
18.19	N-hexadecanoic acid	C_16_H_32_O_2_	256	79.27	
19.15	L-(+)-ascorbic acid 2,6-dihexadecanoate	C_38_H_68_O_8_	652	8.12	
19.83	6-Octadecenoic acid	C_18_H_34_O_2_	282	16.69	
20.04	Octadecanoic acid	C_18_H_36_O_2_	284	57.20	
23.07	1,2-benzenedicarboxylic acid, mono(2-ethylhexyl) ester	C_16_H_22_O_4_	278	5.28	
27.19	Cholesta-5,22-dien-3-ol, (3.beta.)-	C_27_H_44_O	384	2.81	
27.52	Cholesterol	C_27_H_46_O	386	100	
27.88	Ergosta-5,22-dien-3-ol, (3.beta.,22e)-	C_28_H_46_O	398	19.85	
28.50	3beta-hydroxy-5-cholen-24-oic acid	C_24_H_38_O_3_	374	24.26	
28.79	Stigmasterol	C_29_H_48_O	412	4.30	
29.44	Gamma.-sitosterol	C_29_H_50_O	414	20.75	

## Discussion

Bioactive compounds extracted from marine invertebrates, including bivalves, possess broad spectrum antimicrobial activities [22]. Antimicrobial activities have been described in a wide range of molluscan species including oyster (*Crassostrea virginica*), mussel (*Mytilus edulis* and *Geukensia demissa*), muricid mollusks (*Dicathais orbita*) and sea hare (*Dolabella auricularia*) [23-26]. In order to fight against microbial infection, marine mollusks have found the adaptation of antimicrobial factors including antimicrobial compounds and antimicrobial peptides etc. In the present study, the active compounds extracted from the mid polar extraction found to be the broad range of antimicrobial and anticancer activities. The extract effectively controlled the pathogenic bacteria including *P. aeruginosa, V. harveyi, V. parahaemolyticus, A. hydrophila, S. aureus;* the fungi include *A. niger, A. flavus, C. albicans, Fusarium*s p and the shrimp killer virus WSSV. The acetone extract of the winged oyster, *Pteria chinensis* was found to have a broad spectral of inhibiting activity against fish pathogenic bacteria [27]. Defer *et al*. [28] reported the antibacterial and antiviral activities in three bivalve and two gastropod marine molluscs (*Cerastoderma edule*, *Ruditapes philippinarum, Ostrea edulis* and *Buccinum undatum*). The edible bivalve species including *Perna viridis* and *Meretrix casta* showed antifungal activities [29]. Limasset et al. [30] reported the antiviral activity in extracts from mussel *Mytilus edulis*, clam *Mercenaria mercenaria*, and oyster *Crassostrea gigas* against the Tobacco Mosaic Virus (TMV). Li and Traxler [31] reported the antiviral activity in clam *Mya arenaria* aqueous extract against an amphibian virus (LT-1).

Based on the secondary screening, the FV fractions are very effective to control the bacteria, fungi, and virus and also had antitumor activities. The structural characterization of the FV revealed that the eleven fatty acid derivatives were identified as hexadecanoic, L-(+)-ascorbic acid 2,6-dihexadecanoate, 1,2-benzenedicarboxylic acid, mono(2-ethylhexyl) ester, octadecenoic and stigmasterol etc. The previous literatures also evidenced that, the lipid derivatives including hexadecanoic acid, octadecenoic acid, L-(+)-ascorbic acid 2,6-dihexadecanoate, 1,2-benzenedicarboxylic acid, mono (2-ethylhexyl) ester, sigmasterol and γ-sitosterol characterized from plants and marine sources were found to be a powerful antimicrobial, antioxidant and anticancer activities.

The FV which extracted from the mobile phase of H25: EA25% contained all fatty acid derivatives which inhibited the pathogenic bacteria at the rate of 15 mm zone of inhibition. As seen in the previous literatures, the compounds including hexadecanoic acid, octadecenoic acid, stigmasterol and γ-sitosterol may inhibit the pathogenic bacteria. This is supported by several researchers. 9-Octadecanoic acid and hexadecanoic acid identified from neem oil had effectively inhibited the pathogenic bacteria including *Staphylococcus aureus* ATCC No. 25923, *Escherichia coli* ATCC No. 44102 and *Salmonella* sp. ATCC No. 50 041 by *in vitro* antibacterial screening [32]. Abou-Elela et al. [33] proved the antibacterial activity using hexadecanoic acid derivatives from marine sponge, *Spongia officinalis* and the brown algae *Cytosoria compressa* against *S. aureus, S. faecalis, P. aeruginosa* and *E.coli*. L (+)-Ascorbic acid, 2,6-dihexadecanoate were identified from the ethanolic extract of *Dacryodes edulis* had potent antibacterial activity against *S.aureus, E. coli, Streptococcus pneumonia* and *Proteus mirabilis* [34]. 1, 2-Benzenedicarboxylic acid bis(2-ethylhexyl) phthalate isolated from the seaweed, *Sargassum weightii*, have antibacterial effect on anumber of bacteria [35]. Moreover stigmasterol isolated from the aerial part of *Spillanthes acmella* effectively controlled various pathogenic bacteria including methycilin resistant *Staphylococcus aureus* (MRSA) [36]. These fatty acid derivatives identified from *S. glomerata* may inhibit cell wall disruption, transcription and translation which lead to the arrest of protein synthesis.

The antifungal fatty acids naturally insert themselves into the lipid bi-layer of the fungal membranes and physically disturb the membrane, resulting in increased fluidity of the membrane. These elevations in membrane fluidity will cause the release of intracellular components, cytoplasmic disorder and eventually cell disintegration. Carballeira et al. [37] studied the antifungal activity of the fatty acids such as 2,6- hexaecadiynoic acid and 2,6-nonadecadiyenoic acid against *Candida albicans* and *Cryptococcus neoformans* respectively. Stigmasterol and γ –Sitosterolwere identified in many plants including *Spillanthes acmella* inhibited various fungal species including *Candida albicans, C. virusei,* and *C. tropicalis* [36,38]. The fatty acid derivatives may interfere or disintegrate the cell wall or spore wall of the pathogenic fungus leading to the arrest of the germination or growth. In the present study, the lipids identified from the *S. glomerata* extract fraction may inhibit the transcription and translation of the WSSV and that lead to the arrest of viral multiplication. The extracts help to reduce the WSSV load in the shrimps’ haemolymph and other tissues. Due to the inactivation of WSSV or the lowest load in the shrimp haemolymph and other tissues, the survival may have been increased than to the control and heaxane and methanolic extracts treated groups. This has been supported by Velmurugan et al. [39] they proved the antiviral activity by delivering *Psidium guajava* methanolic extract containing 1, 2-Benzenedicarboxylic acid bis (2-ethylhexyl) to *Fenneropenaeus indicus* infected with white spot syndrome virus (WSSV) infected shrimp.

Fatty acids have been reported as a potential group of natural products which modulate tumor cell growth. In the present study, the purified fraction (FV) of *S. glomerata* was found to be controlling the tumor mammary epithelial carcinoma cells at the rate of 83% in the 100 μg/ml and 50% in 100 μg/ml respectively in experimental and control. The fatty acid derivatives including hexadecanoic acid and octadecanoic acid isolated and characterized from the *Ceratonia siliqua* pods essential oil inhibited two tumoral human cell lines HeLa and MCF-7 [40]. 6-Octadecenoic acid characterized from crude methanolic extract of *Calendula officinalis* flower effectively suppressed the human epidermoid larynx carcinoma (Hep-2) cell line [41]. 1,2-Benzenedicarboxylic acid and mono(2-ethylhexyl) ester from the ethanolic extract of *Centratherum punctatum* had cytotoxic effect against Ehrlich Ascites Carcinoma cell lines [42]. Also the immature *Citrus grandis,* Osbeck, fruit extract contains γ-sitosterol and stigmasterol which has cytotoxic effects against human leukaemia cells U937 [43].

## Conclusions

Based on the results of the present study, the fatty acid derivatives including hexadecanoic acid, octadecenoic acid, L-(+)-ascorbic acid 2,6-dihexadecanoate, 1,2-benzenedicarboxylic acid, mono (2-ethylhexyl) ester, stigmasterol and γ-sitosterol isolated and characterized from *S. glomerata* had the ability of controlling the pathogenic bacteria, fungi, virus and antitumor activities. Further study is needed to purify the above said fatty acid derivatives and the study the antimicrobial and anticancer activities individually.
